# Zinc Isotope Ratios as Indicators of Diet and Trophic Level in Arctic Marine Mammals

**DOI:** 10.1371/journal.pone.0152299

**Published:** 2016-03-24

**Authors:** Klervia Jaouen, Paul Szpak, Michael P. Richards

**Affiliations:** 1 Max Planck Institute for Evolutionary Anthropology, Department of Human Evolution, Deutscher Platz 6, 04103, Leipzig, Germany; 2 University of British Columbia, Department of Anthropology, Vancouver Campus, 6303 NW Marine Drive, Vancouver, BC Canada, V6T 1Z1; Stockholm University, SWEDEN

## Abstract

Carbon and nitrogen stable isotope ratios of bone collagen are an established method for dietary reconstruction, but this method is limited by the protein preservation. Zinc (Zn) is found in bioapatite and the isotopic compositions of this element constitute a very promising dietary indicator. The extent of fractionation of Zn isotopes in marine environments, however, remains unknown. We report here on the measurement of zinc, carbon and nitrogen isotopes in 47 marine mammals from the archaeological site of Arvik in the Canadian Arctic. We undertook this study to test and demonstrate the utility of Zn isotopes in recent mammal bone minerals as a dietary indicator by comparing them to other isotopic dietary tracers. We found a correlation between δ^66^Zn values and trophic level for most species, with the exception of walruses, which may be caused by their large seasonal movements. δ^6^Zn values can therefore be used as a dietary indicator in marine ecosystems for both modern and recent mammals.

## Introduction

The main biochemical method of determining the diets of recent and fossil mammals is the measurements of the stable isotope ratios of carbon (C) and nitrogen (N) in bone protein (collagen). Recently, a number of researchers have explored the use of the isotope ratios of novel, ‘non-traditional’ elements in mammal tissues as additional dietary indicators, or as the primary dietary indicator where collagen preservation is poor. For example, Knudson and collaborators [1] suggested that strontium (Sr) stable isotopes could be an indicator of trophic level (TL), and it also appears possible that calcium (Ca) isotopes in bone and dental enamel could indicate dairy consumption and weaning [2–4] or other dietary information [5]. Recently, Martin and collaborators [6,7] demonstrated that magnesium (Mg) isotopes combined with other dietary indicators in dental enamel, such as trace elements and carbon stable isotope ratios, could also be used to reconstruct ancient diets. Their study showed a progressive Mg heavy-isotope enrichment in mammal food webs.

This paper is primarily concerned with the measurements of Zn isotope ratios as a dietary indicator. Previous studies of zinc (Zn) isotopes in human blood highlighted strong differences between vegetarians and omnivores [8,9], whereas the link between diet and blood iron (Fe) isotope compositions is unclear [8,10,11]. Recent findings from modern African food webs also demonstrated the possibility of distinguishing carnivores from herbivores using Zn stable isotope measurements from bones and teeth. Zinc isotopes in bioapatite may therefore provide similar information as N isotopes of bone collagen from terrestrial environments. As it is a protein, however, collagen is sensitive to decay due to time and environmental changes. Zn isotopes could therefore be used to trace past mammal diet when the collagen is not preserved.

**Fig 1:**
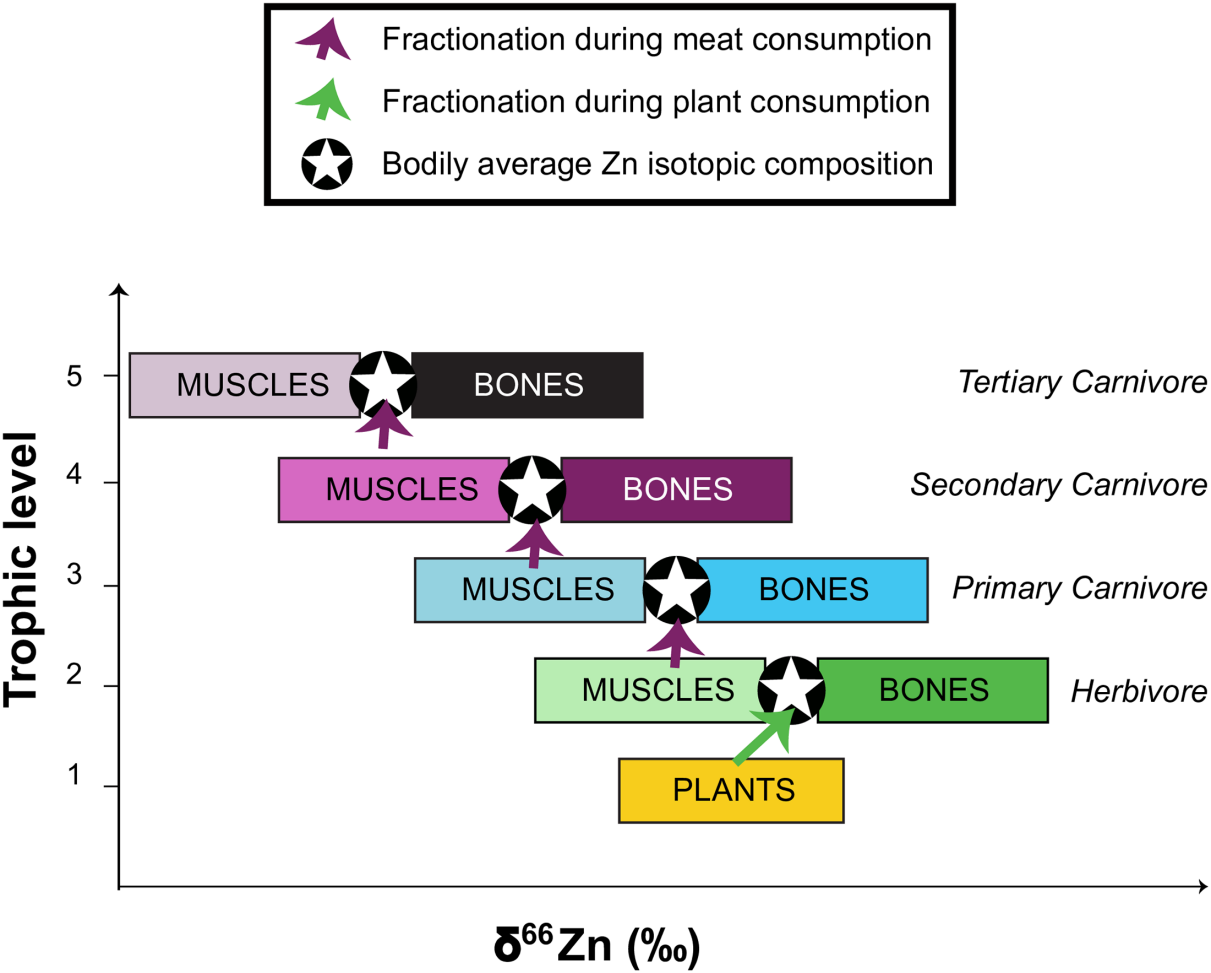
Expected distribution of δ^66^Zn values in animal tissues based on values obtained in modern terrestrial food webs and experimental animals [14,15,16].

Zinc has five stable isotopes (^64^Zn, ^66^Zn, ^67^Zn, ^68^Zn, and ^70^Zn) with respective average natural abundances of 48.6, 27.9, 4.1, 18.8, and 0.6%. As the isotopes 64 and 66 are the most abundant, the ratio of ^66^Zn/^64^Zn expressed as δ^66^Zn is calculated using these two isotopes. Isotopic fractionation occurring during intestinal absorption is one of the two dietary factors impacting the isotopic composition of the Zn in animal tissues. Plant consumption induces a ^66^Zn-enrichment relative to ^64^Zn during intestinal absorption. This enrichment has been attributed to the precipitation of Zn with phytates in the intestine, inhibiting its absorption. This precipitation favors the binding of light Zn isotopes to the phytates, which implies that heavy Zn isotopes are more bioavailable [12,13]. The δ^66^Zn values of herbivore body tissues are therefore higher than the δ^66^Zn values of their diets [13,14] A carnivorous diet does not contain phytates and carnivore body tissues should consequently exhibit less fractionation relative to their food sources. The second dietary factor triggering variability in δ^66^Zn values within the body is the actual Zn isotopic composition of the foods consumed. As an example, muscles are ^66^Zn depleted relative to the global isotopic composition of the body [15,16], and carnivores therefore exhibit lower δ^66^Zn values than their prey [14] (Fig 1). In addition, Costas-Rodriguez et al. [9] reported isotopic composition of various food products (total range ~2 ‰) with a depletion of Zn light isotopes in animal products (milk, eggs, meat and fish) when compared to plant products. As a consequence, we can expect a negative correlation between tissue δ^66^Zn values and trophic levels (Fig 1). Zooplankton δ^66^Zn values also fall within the range of terrestrial animal tissues [17]. Diatoms (phytoplankton) are enriched in Zn heavy isotopes compared to the seawater (δ^66^Zn _seawater_~+0.5‰) to an extent of +0.3‰ [18,19]. The sole exception appears to be for mussels: values reported for their soft tissues show a strong enrichment in Zn heavy isotopes [9,17,20], which could possibly be explained by their bioaccumulator behavior [9]. As a consequence, shellfish consumers, such as walruses, should exhibit relatively high Zn isotopic ratios in their tissues.

The findings reported in Costas-Rodriguez et al. [9], namely that similar values of δ^66^Zn are observed for marine and terrestrial animals, contrast with patterns observed for the isotopic tracers classically employed for dietary reconstruction. C and N isotope compositions are generally higher in marine organisms relative to terrestrial animals. As a result, their consumption can easily be quantified on the basis of tissue C and N isotope compositions. Nitrogen and (to a lesser extent) carbon isotope compositions are also dependent on trophic levels [21–24]. These isotopes are frequently used in marine ecological studies to explore the diet and trophic levels of vertebrate consumers (e.g.[25–28]) and can be used as a basis to better understand how Zn isotopes vary in these environments. For this reason, we expect to see a negative correlation between N and Zn isotopes in food webs, while δ^13^C values can be used to detect additional dietary characteristics such as the ultimate source of primary production (e.g. macroalgae vs. phytoplankton), and benthic vs. pelagic food sources, as benthic organisms generally exhibit higher isotopic ratios [25,26].

**Fig 2:**
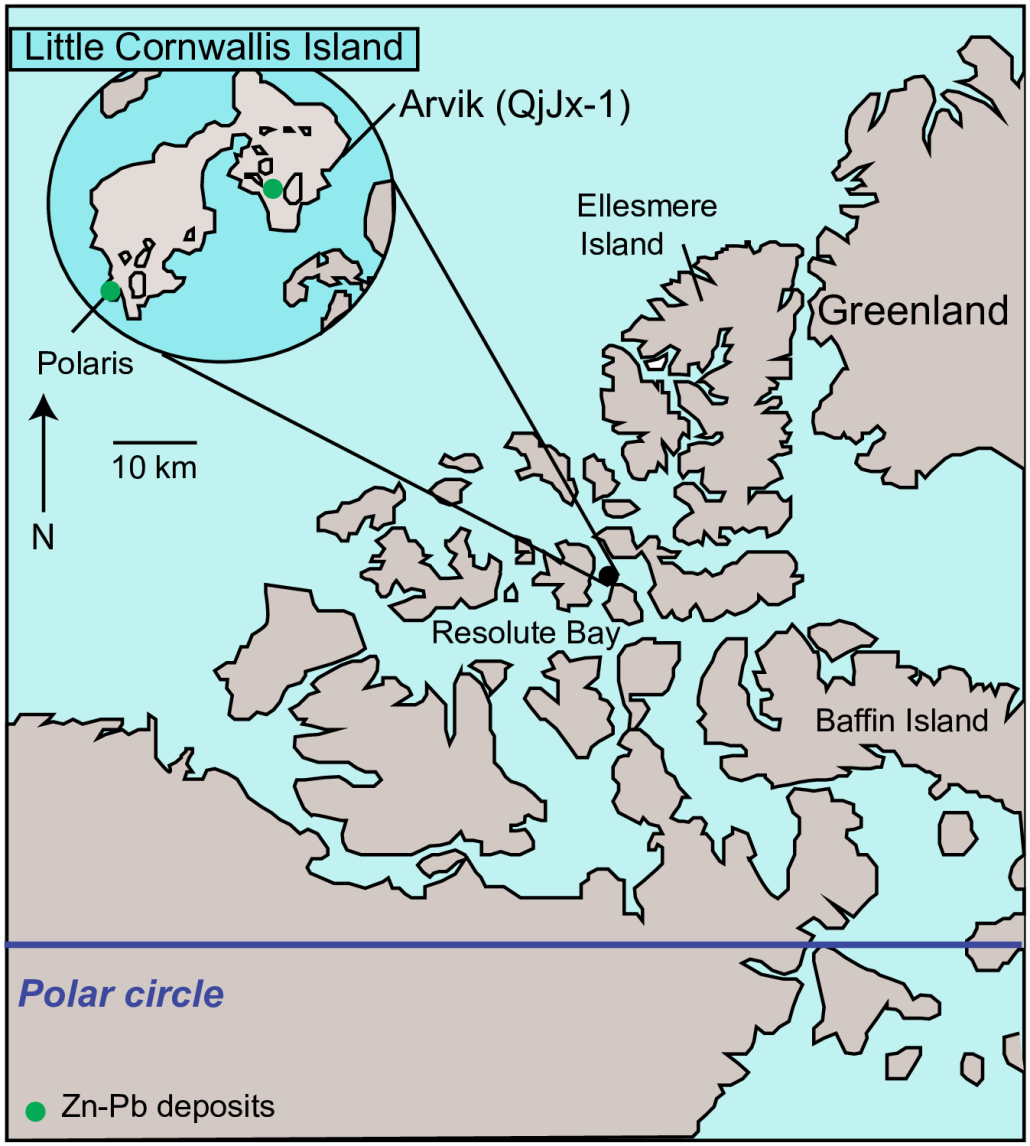
Map showing the location of the site, Zn-Pb deposits (green circles) and the Polaris mine. The map is adapted from the one of Le Moine et al [29].

In the present work, we report bone δ^66^Zn, δ^15^N and δ^13^C values for different animals from the archeological site of Arvik on Little Cornwallis Island, Nunavut, Canada (Fig 2). We explore the link between Zn isotopic composition and diet in a marine food web. Because the effect of metabolism on δ^66^Zn values of the body is not known for non-mammal species, we chose to focus on mammals. We studied four species with different trophic levels: 1) walruses (*Odobenus rosmarus*), which mostly feed on mollusks and other benthic invertebrates (TL~3.0); 2) bearded seals (*Erignathus barbatus)*, which feed on benthic invertebrates and fish (TL~3.5); 3) ringed seals (*Pusa hispida)*, whose diet relies on fish, but also includes large zooplankton and benthic invertebrates (TL~4.0); 4) polar bears (*Ursus maritimus)*, tertiary carnivores which are specialized seal hunters (TL~5.0).

## Materials and Methods

### Materials

We analysed samples from 47 adult individuals from four mammal species: bearded seals (*n* = 13), ringed seals (*n* = 12), polar bears (*n* = 10) and walruses (*n* = 12). Information on the anatomical element and archaeological context of the bones is reported in the Table A in S1 File. All samples come from the archeological site Arvik (QjJx-1), located in the northeast of Little Cornwallis Island in the Canadian Central High Arctic (Fig 2). The site, located on a fossil beach, 5–8 m above sea level, is known for its Dorset occupation [29]. The bones were found on the surface and the subsurface of the site and are characterized by outstanding preservation (S1 File). Two radiocarbon dates have been obtained from this site with calibrated dates of A.D. 429–665 and A.D. 347–604 (2σ range) [29,30].

### Ethics Statement

All relevant permissions were obtained for this study. Permission to sample the materials was obtained from the Prince of Wales Northern Heritage Centre, Yellowknife, YT, where the materials are curated. A permit for destructive analysis was obtained from the Culture and Heritage Division, Culture, Language, Elders & Youth, Government of Nunavut (#90–682) issued in March 2014.

### Carbon and nitrogen analytical technique

Bones were mechanically cleaned by abrasion with a diamond-tip burr and subsequently ~250 mg of material was removed using a diamond-tip cutting wheel. These bone samples were demineralized in 0.5 M HCl at 4°C for a period of several weeks. After demineralization, the remaining insoluble collagen was solubilized in 10^−3^ M HCl at 75°C for 48 h. The solution containing the soluble collagen was then filtered using a 60–90 μm filter (Elkay, Hampshire, UK) to remove insoluble particulates and then filtered using 30 kDa molecular weight cut-off centrifuge tubes (Pall Corporation, Port Washington, NY). The >30 kDa fraction was then frozen and lyophilized. Elemental and isotopic compositions of the dried collagen were determined in duplicate at the University of British Columbia using an Elementar Isoprime continuous-flow isotope ratio mass spectrometer coupled to a Vario Micro elemental analyzer (Hanau, Germany). Details on calibration, analytical accuracy and precision are presented in S2 File).

### Zinc analytical technique

Bone chunks were sampled from the inner part of massive bones, except for some walruses for which only skulls were available. The chunks were abraded using a dremel equipped with a diamond drill to remove the cancellous parts, which are likely to retain sediment particles. For some walruses, no cortical part was available. For these animals, cancellous parts were analysed to investigate the potential effect of soil contamination. Samples of bone (7 to 90 mg) were dissolved in 1mL of double-distilled HCl 7.0 N+0. 001% H_2_O_2_, then evaporated and redissolved in HBr 1.5 N. Zn was purified in two steps using first the modified technique adapted from Moynier et al. [31] first described in Jaouen et al [14]. For this protocol, Zn is purified on 1 mL AG-1x8 resin (200–400 mesh) using 2mL of HBr 1.5 N for matrix residue elution and 5 mL of HNO_3_ 0.5 N for Zn elution. Every preparation batch included at least one standard (in-house or reference material) and a blank. Column steps allow the quantitative recovery of the initial amount of Zn [20,31]. Zinc concentrations were estimated using a regression equation based on the ^64^Zn signal intensity (V) of three solutions with known concentrations (150, 300 and 600 ppb), following a protocol adapted from the one used for Sr by Copeland et al. [32]. Zinc isotopic ratios were measured using the protocol of Toutain et al. [33] and Cu doping on a Thermo Neptune Multicollector ICP-MS at the Max Planck Institute for Evolutionary Anthropology. The in-house standard Zn AA-MPI was used for standard bracketing. Isotopic ratios were corrected so that the results are given relative to the international standard JMC-Lyon.

### Diagenesis test

There is no mineralogical criterion for unambiguous detection of diagenesis [34]. Here, we use combined measurements of Zn concentration and stable isotope composition in different fractions of the walruses' bones to evaluate whether the initial biological metal inventory has been perturbed by a diagenetic end-member. For any given element, the addition of variable proportions of a soil component to the initial biogenic inventory is expected to produce a mixing line, i.e., a correlation between concentrations and isotopic compositions [35]. Values obtained for cancellous parts of the bones (more likely to retain soil particles because of greater porosity relative to cortical bone) and for the cortical parts were compared within and among individuals.

### Statistical analyses

In order to detect significant differences between isotopic values for each species, we conducted a Kruskal-Wallis test followed by the Nemenyi post-hoc test. To assess the differences between cancellous and cortical bone values in walruses, we performed a two-paired Wilcoxon test. These non-parametric tests, performed using R, can be applied when data do not follow a normal distribution [36].

## Results

### Data quality control

#### Sample preservation

**Fig 3:**
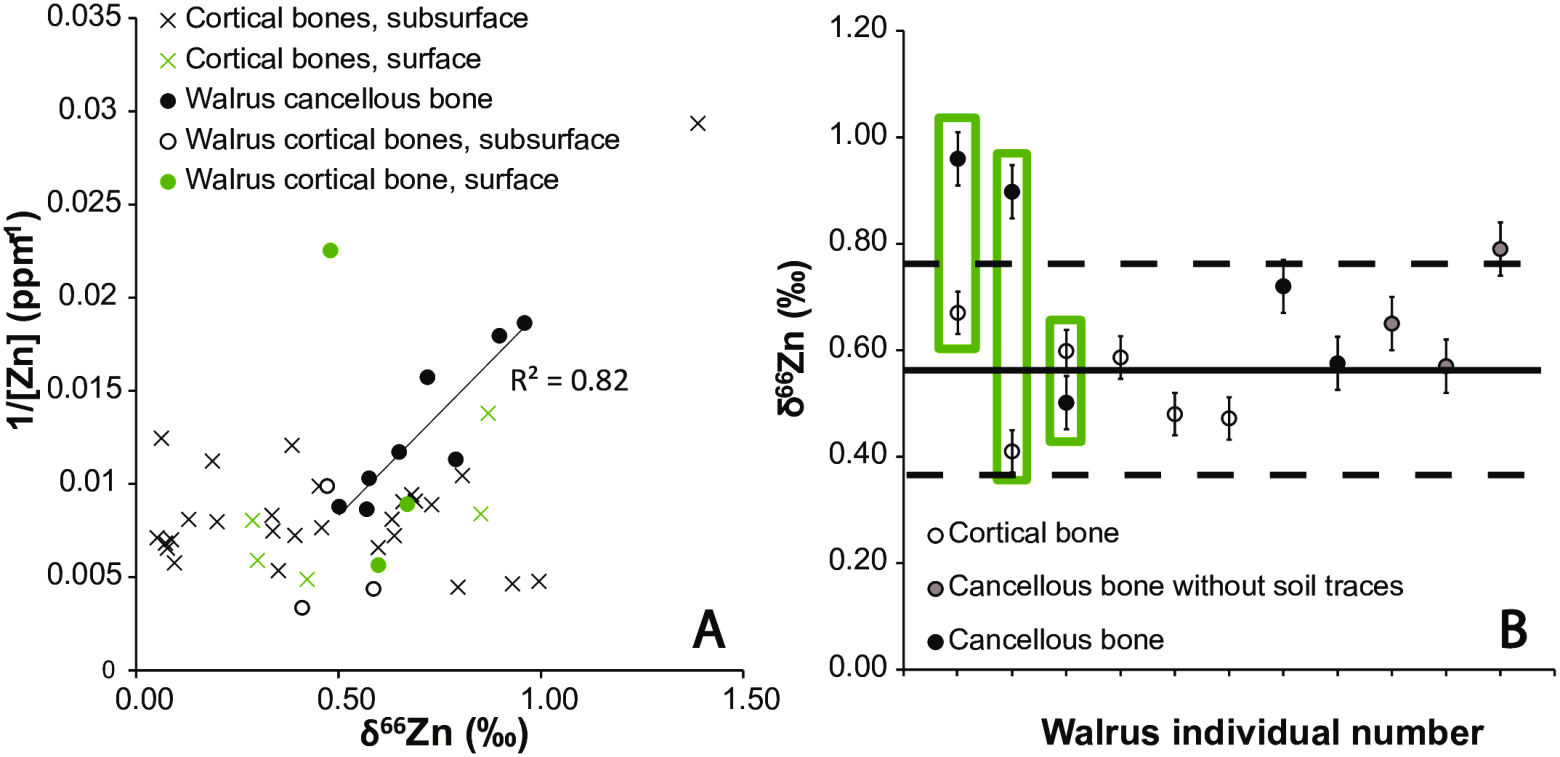
A. Relationship between the Zn concentration and δ^66^Zn values of cancellous (black circles) and cortical part of bones of walruses (circles) and other mammals (crosses), found in surface (green colors) and subsurface deposits (black colors). All the cancellous bone samples fall on a regression line. B. δ^66^Zn values of cancellous and cortical fractions of walrus bones. Lines define the average and two standard deviation area of cortical values. Green rectangles frame values for same individuals.

Zinc concentrations in the cortical and cancellous parts of the bones are consistent with mammal physiological levels, usually ranging between 20 and 350 ppm [14,15,37–39]. Concentrations and isotopic ratios are not significantly correlated in cortical bone but the correlation clearly appears in cancellous bone (Fig 3A). The fact that cancellous parts of bones tend to have the δ^66^Zn values suggests that a diagenetic component enriched in heavy Zn isotopes filled the bone alveoli but did not penetrate the cortical part. An alternative explanation would be that Zn from the cancellous part was leached out, which would explain the lowest Zn concentration of the cancellous part of the bones. The cortical part of the bones seems to be preserved from these processes, with one exception: one bearded seal δ^66^Zn value (Individual 4806, Supporting Information) was an outlier and may fall on the regression line found for the cancellous bones (Fig 3A). This value may not reflect the biogenic one. All the other δ^66^Zn values correspond to the values in the living individuals. For this reason, we use only cortical bone values for the interpretation of Zn isotopic bone signatures. Even in exceptionally well-preserved archaeological bones (S1 File), only the very dense cortical bone consistently provides an endogenous signature.

#### Analytical precision

δ^66^Zn uncertainties were estimated from standard replicate analyses and ranged between ±0.04 ‰ and ±0.06 ‰. Duplicates were performed for several samples, with a standard deviation ranging between ±0.02 ‰ and ±0.04 ‰. The standard reference material SRM-1486 (bone ash) and in-house standards (AZE, cow bone powder) were analysed along with the samples. Obtained values correspond to those published elsewhere (Table B in S1 File). δ^66^Zn, δ^67^Zn and δ^68^Zn values obtained for all samples and standards measured in this study lie on a line with a slope in agreement with the theoretical value.

### Data description

**Fig 4:**
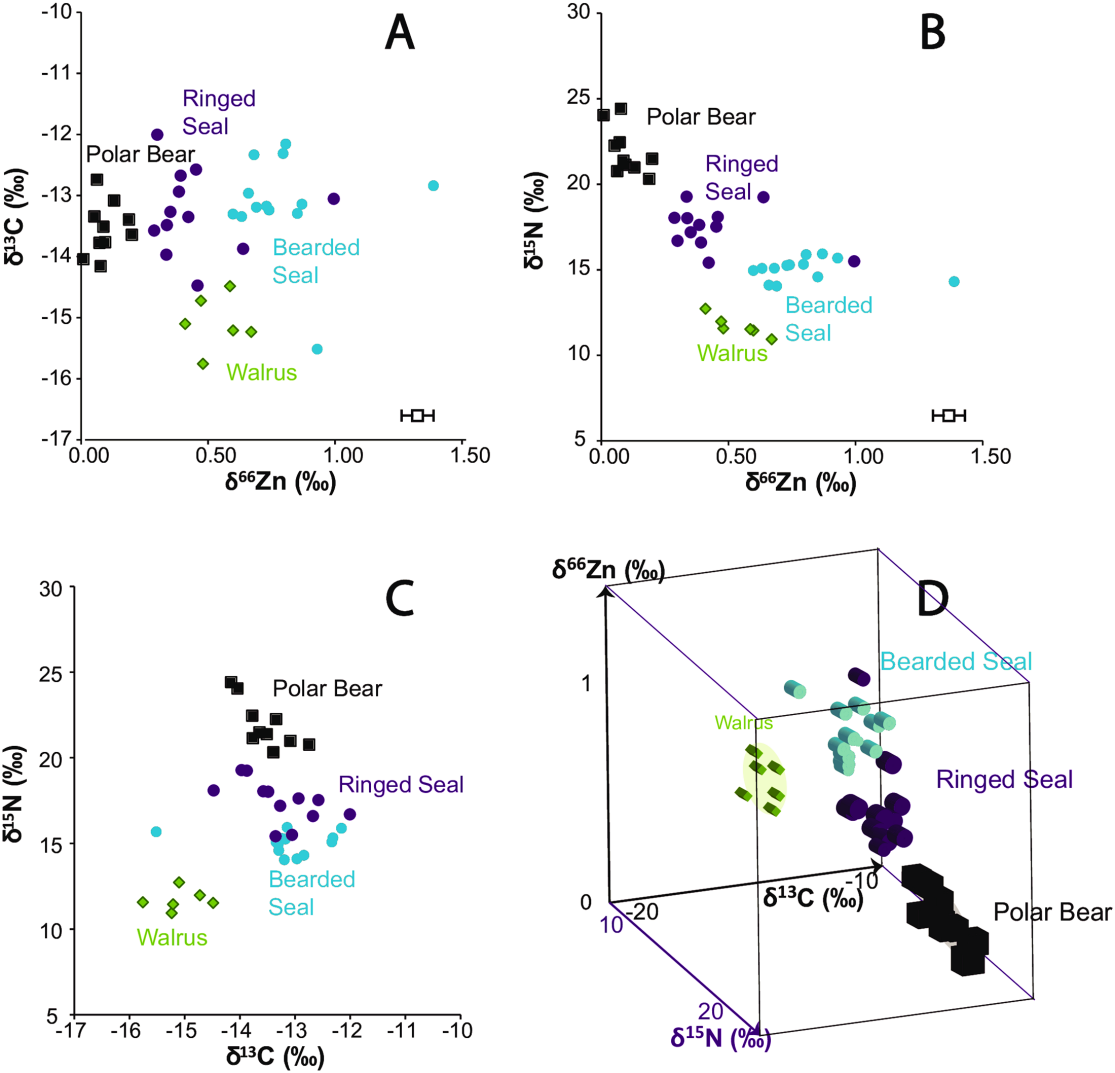
Cortical bone isotopic values of arctic mammals for C, N, and Zn. *A*) δ^66^Zn_bioapatite_ vs. δ^13^C_collagen_ values for four species of marine mammals (B) δ^66^Zn_bioapatite_ vs. δ^15^N_collagen_ values, (*C*) δ^15^N_collage_ vs. δ^13^C_collagen_ and (*D*) 3D plot showing that Zn, N and C isotopic compositions of marine mammals enables a distinguishing of the different species. Measurement uncertainties are given by the white boxes.

The complete dataset of C, N and Zn isotopic compositions is reported in Table A in S1 File), and a synthetic overview containing statistical results is given in Table 1. The total range of measured values for δ^66^Zn in bone bioapatite was 1.38 ‰ (from +0.01 ‰ to +1.39 ‰). The range in bone collagen was 3.76 ‰ for C isotopes (from −15.76 ‰ to −12.00 ‰) and +13.56 ‰ for N isotopes (from +10.93 ‰ to +24.49 ‰) (Table 1). The highest Zn isotopic ratios were measured in seal bones, contrasting with the low values exhibited by the polar bears (Fig 4, Table 1). Polar bears exhibited the highest δ^15^N values. Walruses had the lowest δ^13^C values. They were also characterized by the lowest N isotopic ratios, but the δ^66^Zn values of their bones overlapped with those of ringed and bearded seals. In comparison to the cortical part of the bones, Zn isotopic and concentration values were respectively higher and lower in the cancellous relative to the cortical part of walrus bones, even if the difference was not significant for the concentrations (Fig 3, Wilcoxon test, δ^66^Zn: p = 0.03, [Zn]: p = 0.09).

**Table 1:** Descriptive statistics for C, N and Zn stable isotopes. SD = standard deviation, n = number of samples, TL = trophic level.

		**δ**^**66**^**Zn**				
	**TL**	N	Mean	Min	Max	SD
All (crtical)		41	+0.49	+0.01	+1.39	0.31
Polar Bears	5	12	+0.10	+0.01	+0.20	0.06
Ringed Seals	4	12	+0.45	+0.29	+1.00	0.19
Bearded Seals	3.5	13	+0.80	+0.60	+1.39	0.20
Walruses (cortical bone)	3	6	+0.54	+0.41	+0.67	0.10
Walruses (spongious bone)	5	8	+0.71	+0.50	+0.96	0.16
		**δ**^**13**^**C**				
		N	Mean	Min	Max	SD
All		46	−13.72	−15.76	−12.00	0.91
Polar Bears	5	12	−13.27	−14.16	−12.74	0.43
Ringed Seals	4	10	−13.54	−14.47	−12.00	0.68
Bearded Seals	3.5	13	−13.14	−15.51	−12.16	0.82
Walruses	3	11	−15.07	−15.76	−14.48	0.46
		N	Mean	Min	Max	SD
All		46	+16.31	+10.93	+24.41	3.51
Polar Bears	5	12	+21.92	+20.32	+24.41	1.37
Ringed Seals	4	10	+17.43	+15.42	+19.27	1.24
Bearded Seals	3.5	13	+15.04	+14.04	+15.93	0.63
Walruses	3	11	+11.48	+10.93	+12.72	0.49

### Statistical differences between species

Results of the Kruskal Wallis tests are given in the Table C in S1 File. The results indicated that the performance of the comparisons was not similar between the groups of isotope values. We were therefore able to conduct the post-hoc Nemenyi test (Table D in S1 File). The δ^13^C values of the walruses were significantly lower relative to both ringed and bearded seals, but not polar bears. Nevertheless, polar bear and walrus δ^13^C values do not overlap (Fig 4, Table D in S1 File). The δ^66^Zn values of the polar bears were clearly distinct relative to the other species. The walrus values for Zn isotopes were similar to both seal species, but bearded seals had significantly lower δ^66^Zn values than the ringed seals (Fig 4, Table D in S1 File). The walruses exhibited significantly lower δ^15^N values compared to the highest trophic level animals (polar bears and ringed seals).

## Discussion

Carbon and N bone isotopic ratios are consistent with values expected based on previous studies [28,40,41]. The N isotopic ratios that we report are perfectly consistent with the trophic level of the four species (Fig 4). A slight positive δ^13^C shift with trophic level (0 to 2 ‰) is sometimes observed within a single food web [42], but polar bears do not exhibit higher δ^13^C values than ringed or bearded seals (Fig 4). The fact that polar bears have significantly lower C isotopic compositions than bearded seals indicates that they probably mostly fed on ringed seals, which is consistent with the numerical and biomass composition of their prey as well as fatty acid analyses previously reported in the circumpolar range [43–46]. That polar bear δ^13^C values are not significantly higher than ringed seal δ^13^C values may be due to a high proportion of juvenile ringed seals in the diet, which tend to have lower tissue δ^13^C values in comparison to adults [47]. Ringed seals show the highest dispersion of δ^13^C values, which can be explained by the fact that they feed on both pelagic and benthic organisms, whereas bearded seals mostly rely on benthic prey. Benthic organisms are known to exhibit higher C isotopic ratios [25]. Surprisingly, walruses have low bone collagen δ^13^C values, which is inconsistent with their benthic invertebrate-dominated diet (Fig 4). A possible explanation could be linked to the mobility of the different species. Bearded and ringed seals are year-round residents, while walruses migrate seasonally [48–50]. Little Cornwallis Island is located near the western edge of the Atlantic walrus’ (*Odobenus rosmarus rosmarus*) present range, and Pacific walruses (*Odobenus rosmarus divergens*) do not appear to venture east into the Canadian Beaufort Sea [51]. The walruses deposited at Arvik likely fed in another location, further to the east, for a portion of the year. A study conducted in the western North American Arctic reported a decline in δ^13^C values of zooplankton from west to east [52], suggesting that regional differences in consumer δ^13^C values is likely.

δ^66^Zn values show a different picture from the other proxies. As a Zn light isotope enrichment is expected within a food web (Fig 1), one can also expect a negative correlation between δ^66^Zn and δ^15^N values since N isotopic ratios are dependent on the trophic level. It seems that this correlation exists if either walruses or bearded seals are excluded (Fig 4 and Figure A in S1 File). As mentioned before, bearded seals show similar δ^13^C values relative to polar bears and ringed seals, but walruses are isotopically depleted. Intensive mollusk consumption by walruses cannot account for this pattern as initial results have demonstrated that mussels are strongly enriched in Zn heavy isotopes [9]. Walruses can also occasionally feed on seals or birds [53–55], but this behavior does not appear to be quantitatively significant for the Arvik walruses as the δ^15^N values are not higher than expected. Given that walruses are specialized benthic feeders [56] and benthic organisms have higher δ^13^C values than pelagic organisms [25,26], the fact that they have δ^13^C values that are significantly lower than ringed or bearded seals suggests that they spent a considerable amount of their time foraging in another, isotopically distinct location. Significant variation of surface water δ^66^Zn values has been documented [57] and as was the case with the unusual δ^13^C values for walruses, their δ^66^Zn values may differ from the expected patterns relative to ringed and bearded seals because they foraged in a different location. Further support for this notion comes from the fact that walrus δ^15^N and δ^66^Zn values are negatively correlated, suggesting the relationship between these two isotopes does occur in walruses but may be mediated by regional effects. Additional studies examining geographic variation in consumer δ^66^Zn values are necessary to clarify this issue.

The correlation between δ^15^N and δ^66^Zn is difficult to discuss within a species, as the sample size and the range in isotopic compositions for each group is relatively small. Still, a trend appears for polar bears and walruses, but not for the seals. Previous studies on archeological human bones and human blood did not find any correlation between δ^15^N and δ^66^Zn values [58,59]. The explanation may be that both humans and seals include multiple food sources in their diet, whereas polar bears and walruses tend to exhibit a more specialized and homogenous diet. In much the same way that C and N isotopic compositions are influenced by local environmental conditions [60,61], the Zn isotopic compositions of a food web may also be dependent on its location [13,14]. As a consequence, the trophic level effect on the Zn isotopic compositions of animal tissues may be concealed when different food webs are considered together.

## Conclusions

The results of this study show that the δ^66^Zn values of marine mammal bones are strongly affected by animal diet but also depend on the environment. Nitrogen and Zn isotopic compositions were negatively correlated but walruses did not fall on the regression line. By combining these analyses with classic isotopic dietary tracers, we realised that this pattern could be explained by the mobility of walruses, which would not necessarily belong to the same food web for some or most of the year. Unlike C and N isotopes, the range of δ^66^Zn values observed in terrestrial and marine mammals is similar. Thus, δ^66^Zn adds complementary information to other dietary indicators such as δ^13^C. Future dietary and trophic ecology applications might, however, require working on low mobility individuals as a provenance factor is suspected in the Zn isotopic signature of bone. On the other hand, the δ^66^Zn values could be an additional provenance indicator to complement C and N isotope measurements in marine environments.

## Supporting Information

S1 File **Figure A**. Relationship between Zn isotopic compositions of terrestrial and marine mammal bones and trophic levels. **Table A.** Location, type of samples, species, collagen preservation, concentrations (C, N, Zn) and isotopic compositions (C, N, Zn) of the different marine mammal samples analyzed in the study. **Table B.** δ^66^Zn values of in house standard and reference material. Zn delta values are corrected for the standard JMC Lyon. **Table C.** Results of the Kruskal-Wallis test (χ2 and p values) performed on the isotopic values (C, N and Zn) for the different species. **Table D.** Matrix of the p-values resulting from the Nemenyi test comparing the isotope compositions of the different species.* p<0.05, ** p<0.005, ***p<0.0005
(PDF)

S2 File **Table A.** Standard reference materials used for calibration of δ^13^C relative to VPDB and δ^15^N relative to AIR. **Table B.** Standard reference materials used to monitor internal accuracy and precision. **Table C**. Accuracy and precision of calibration and check standards for each analytical. **Table D.** Accuracy and precision of calibration and check standards for all analytical sessions (cumulative). **Table E.** Duplicate sample carbon and nitrogen isotopic compositions and absolute difference between measurements.
(PDF)
