# Supplementary material for: Zinc Isotope Ratios as Indicators of Diet and Trophic Level in Arctic Marine Mammals
Source: PLoS One. 2016 Mar 24;11(3):e0152299. doi: 10.1371/journal.pone.0152299 (PMC4806842; doi:10.1371/journal.pone.0152299)
Supplement: S1 File — Figure A. Relationship between Zn isotopic compositions of terrestrial and marine mammal bones and trophic levels. Table A. Location, type of samples, species, collagen preservation, concentrations (C, N, Zn) and isotopic compositions (C, N, Zn) of the different marine mammal samples analyzed in the study. Table B. δ66Zn values of in house standard and reference material. Zn delta values are corrected for the standard JMC Lyon. Table C. Results of the Kruskal-Wallis test (χ2 and p values) performed on the isotopic values (C, N and Zn) for the different species. Table D. Matrix of the p-values resulting from the Nemenyi test comparing the isotope compositions of the different species.* p<0.05, ** p<0.005, ***p<0.0005 (PDF) [file pone.0152299.s001.pdf]

Supporting Information 1
Dataset, additional discussion and statistics

Environmental context

The geology of Little Cornwallis Island is characterized by Paleozoic marine carbonates, and Arvik bedrock is made of Ordovician wackestone and limestone [1]. Some Zn-Pb deposits are present in other parts of the island ([1], and Figure 2). Ice caps surround Arvik, which is also located close to the sea (Figure 2). Zinc concentrations in snow are below 70 ppb during preindustrial times [2,3] but can reach 2 ppm in modern snow [3]. In the deep oceans [4–6], these concentrations are usually around 5nM, but can be much lower in the photic zone [5,7]. This strong depletion is attributed to biological uptake [8]. The δ^{66}Zn values of oceans are very homogeneous, but it has been shown that a horizon at 40-80 m – where Zn regeneration happens − tends to be isotopically lighter ($\Delta Zn_{surface-horizon}$≈-0.3‰, [9–11]). Nevertheless, this observation is valid for the open sea whereas coastal Zn isotopic compositions are unknown [12]. Significant variation of the surface water δ^{66}Zn values have also been documented [11].

Soil contamination:

The marine mammal bones from our study lied on or below the surface for more than 1000 years, according to two calibrated dates: A.D. 429-665 and A.D. 347-604 (2σ range) [13,14]. Since the 6thcentury, thaws were likely to happen, especially during the Medieval Warm Period, and water may have entered the bones. Moore et al., 14] documented the evolution of the temperature using varved sediment from the Donard

Lake of the Baffin Island (Figure 2) found evidence for a period of elevated summer temperatures extending from 1200 to 1375 AD, followed by cooler conditions from 1375 to 1820 AD. It should be noted, however, that the temperatures during the 13th–14th centuries were only one degree higher than nowadays [15]. Because the soil of the island is a continuous permafrost [16] and the temperatures in Little Cornwallis Island are lower than in southern Baffin Island, soil contamination of the bones is expected to be extremely limited.

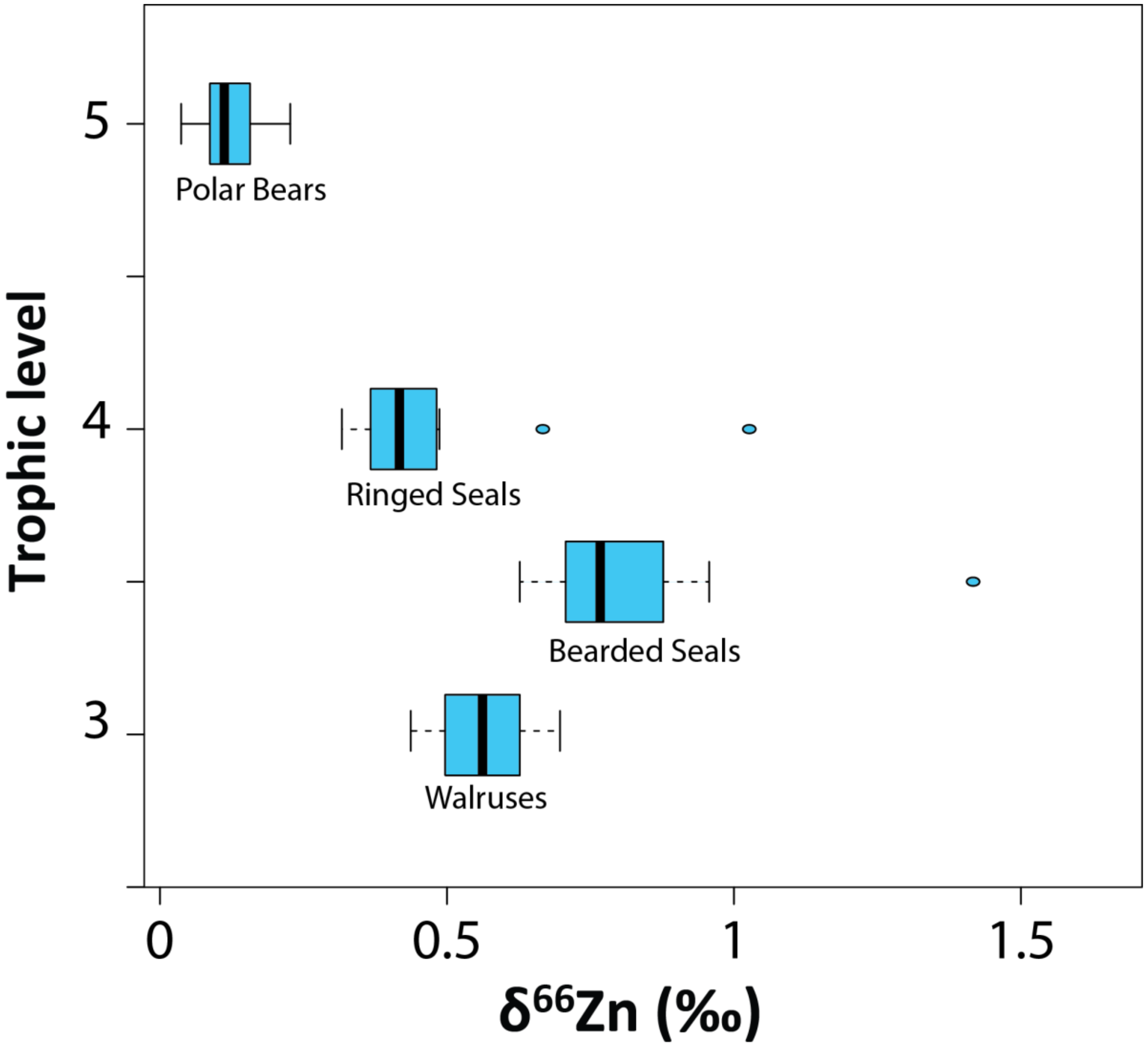

Figure A. Relationship between Zn isotopic compositions of terrestrial and marine mammal bones and trophic levels.

Table A: Location, type of samples, species, collagen preservation, concentrations (C, N, Zn) and isotopic compositions (C, N, Zn) of the different marine mammal samples analyzed in the study.

Id	% Collagen	Site	Feature	Unit	Sample Location	Species	Sampled bone description	$\delta^{13}C$ (‰)	$\delta^{15}N$ (‰)	%C	%N	C/N	$\delta^{66}Zn_{JMC}$ (‰)	$\delta^{67}Zn_{JMC}$ (‰)	$\delta^{68}Zn_{JMC}$ (‰)	[Zn] ppm
4695	18,8	QjJx-1	Feature 49	445N/504E NW	Subsurface	Ringed seal	cortical	-13,87	19,24	43,03	14,89	3,37	0,64	0,93	1,23	139
4696	18,2	QjJx-1	Feature 49	445N/506E NW	Subsurface	Polar bear	cortical	-13,64	21,49	42,12	14,99	3,28	0,20	0,38	0,41	125
4702	18,4	QjJx-1	Feature 49	445N/503E SE	Subsurface	Polar bear	cortical	-13,76	21,14	41,82	14,43	3,38	0,09	0,16	0,20	173
4704	17,6	QjJx-1	Feature 49	449N/501E NW	Subsurface	Bearded seal	cortical	-15,51	15,68	45,77	14,04	3,41	0,93	2,03	2,14	216
4711	12,9	QjJx-1	Feature 20	554N/533E	Surface	Walrus	spongious	-15,23	10,92	40,00	14,33	3,25	0,96	1,25	1,72	54
4711	12,9	QjJx-1	Feature 20	554N/533E	Surface	Walrus	cortical powder	-15,23	10,93	40,40	14,38	3,28	0,67	0,95	1,29	112
4717	19,2	QjJx-1	Feature 20	554N/533E NW	Subsurface	Polar bear	cortical	-13,78	22,45	42,67	15,05	3,31	0,07	0,19	0,22	147
4719	15,2	QjJx-1	Feature 20	553N/532E NE	Subsurface	Walrus	spongious	-15,72	11,29	41,71	14,02	3,47	0,72	1,19	1,47	64
4721	14,7	QjJx-1	Feature 12	599N/527E SE	Surface	Ringed seal	cortical	-13,57	18,04	41,26	14,51	3,32	0,29	0,43	0,60	124
4722	16,8	QjJx-1	Feature 12	600N/527E SE	Subsurface	Polar bear	cortical, trace of spongious	-14,16	24,41	43,35	14,90	3,39	0,08	0,17	0,19	153
4733	15,7	QjJx-1	Feature 74	377N/522E NW	Surface	Ringed seal	cortical	-12,00	16,69	42,64	15,22	3,27	0,30	0,29	0,55	170
4735	14,2	QjJx-1	Feature 74	379N/522E SW	Subsurface	Bearded seal	cortical	-13,19	14,04	42,54	15,17	3,27	0,69	1,01	1,35	110
4749	20,5	QjJx-1	Feature 10	607N/517E SW	Surface	Walrus	bit spongious but very clean	-14,93	11,21	40,14	14,10	3,32	0,65	1,26	1,47	85
4753	16,1	QjJx-1	Feature 10	610N/516E NE	Subsurface	Bearded seal	cortical	-13,34	15,08	42,80	15,30	3,26	0,63	0,99	1,29	123
4755	19,4	QjJx-1	Feature 10	612N/516E SE	Subsurface	Polar bear	cortical	-13,51	21,38	41,87	14,57	3,35	0,09	0,23	0,21	143
4757	15,3	QjJx-1	Feature 10	611N/517E NE	Surface	Bearded seal	cortical	-13,14	15,93	41,20	14,40	3,34	0,87	1,45	1,73	73
4766	19,3	QjJx-1	Feature 10	611N/517E SW	Subsurface	Polar bear	cortical	-13,39	20,32	42,27	14,97	3,29	0,19	0,27	0,37	89
4767	12,5	QjJx-1	Feature 10	611N/517E SW	Subsurface	Ringed seal	cortical	-13,48	18,02	42,97	15,18	3,30	0,34	0,52	0,71	134
4768	13,4	QjJx-1	Feature 10	611N/517E SW	Subsurface	Ringed seal	cortical	-13,27	17,19	43,75	15,40	3,31	0,35	0,65	0,79	187
4773	16,5	QjJx-1	Feature 3	600N/573E NW	Subsurface	Polar bear	cortical	-12,74	20,76	43,64	15,49	3,29	0,06	0,10	0,08	80
4774	15,8	QjJx-1	Feature 3	603N/537E SE	Subsurface	Walrus	spongious	-15,10	12,72	40,24	14,04	3,34	0,90	1,22	1,58	56
4774	15,8	QjJx-1	Feature 3	603N/537E SE	Subsurface	Walrus	cortical powder	-15,10	12,72	40,24	14,04	3,34	0,41	0,89	1,08	298
4775	11,5	QjJx-1	Feature 3	603N/537E NW	Surface	Ringed seal	cortical	-13,35	15,42	41,72	15,24	3,19	0,42	0,59	0,86	205
4776	15,2	QjJx-1	Feature 3	603N/537E SE	Subsurface	Bearded seal	cortical	-12,31	15,32	42,52	15,18	3,27	0,80	1,22	1,60	224

Id	% Collagen	Site	Feature	Unit	Sample Location	Species	Sampled bone description	$\delta^{13}C$ (‰)	$\delta^{15}N$ (‰)	%C	%N	C/N	$\delta^{66}Zn_{JMC}$ (‰)	$\delta^{67}Zn_{JMC}$ (‰)	$\delta^{68}Zn_{JMC}$ (‰)	[Zn] ppm
4787	14,9	QjJx-1	Feature 3	599N/536E SW	Subsurface	Ringed seal	cortical	-12,94	17,62	46,17	15,98	3,37	0,39	0,62	0,80	83
4801	14,1	QjJx-1	Feature 9	612N/531E NW	Subsurface	Ringed seal	cortical	-12,58	17,53	46,15	16,47	3,27	0,45	0,70	0,89	101
4806	16,3	QjJx-1	Feature 74	379N/522E SE	Subsurface	Bearded seal	cortical	-12,84	14,31	44,83	15,79	3,31	1,39	2,08	2,71	34
4808	20,0	QjJx-1	Feature 74	379N/522E SE	Subsurface	Walrus	bit spongious	-15,50	11,27	42,61	14,46	3,44	0,58	1,30	1,44	97
4810	16,6	QjJx-1	Feature 74	379N/522E NE	Subsurface	Bearded seal	cortical	-12,33	15,09	44,72	15,69	3,33	0,68	0,98	1,38	106
4811	9,6	QjJx-1	Feature 74	379N/522E NE	Subsurface	Ringed seal	cortical	-13,05	15,49	44,37	15,42	3,36	1,00	1,69	1,99	210
4817	11,7	QjJx-1	Feature 9	608N/529E SE	Subsurface	Ringed seal	cortical	-12,68	16,59	45,35	16,16	3,27	0,39	0,56	0,82	138
4823	15,3	QjJx-1	Feature 9	610N/531E SW	Subsurface	Bearded seal	cortical	-12,96	14,10	46,55	16,77	3,24	0,66	1,06	1,30	111
4828	15,0	QjJx-1	Feature 9	612N/531E NE	Subsurface	Bearded seal	cortical	-13,30	14,95	47,19	16,81	3,27	0,60	1,02	1,26	152
4829	18,5	QjJx-1	Feature 10	609N/517E SE	Subsurface	Bearded seal	cortical	-13,17	15,25	46,44	16,43	3,30	0,73	1,14	1,46	113
4835	17,8	QjJx-1	Feature 10	609N/517E SW	Subsurface	Bearded seal	cortical	-12,16	15,89	44,86	15,78	3,32	0,81	1,21	1,54	96
4846	17,6	QjJx-1	Feature 16	575N/534E NW	Subsurface	Ringed seal	cortical	-13,97	19,27	42,66	14,55	3,42	0,34	0,54	0,67	120
4849	27,3	QjJx-1	Feature 16	574N/537E NE	Surface	Walrus	spongious	-15,21	11,45	42,11	14,42	3,41	0,50	0,75	1,04	114
4849	27,3	QjJx-1	Feature 16	574N/537E NE	Surface	Walrus	cortical	-15,21	11,45	42,11	14,42	3,41	0,60	0,89	1,19	177
4861	19,9	QjJx-1	Feature 16	573N/535E SW	Subsurface	Walrus	cortical	-14,48	11,53	43,49	15,66	3,24	0,59	0,91	1,16	229
4864	18,9	QjJx-1	Feature 16	573N/536E SW	Surface	Walrus	cortical	-15,76	11,56	43,73	14,22	3,43	0,48	0,71	1,02	44
4871	16,9	QjJx-1	Feature 3	600N/537E SW	Subsurface	Walrus	cortical	-14,72	11,98	40,97	14,20	3,36	0,47	0,74	0,97	101
4875	17,0	QjJx-1	Feature 24			Polar bear	cortical	-14,04	24,05	42,88	14,98	3,34	0,01	0,09	0,08	172
4884	18,8	QjJx-1	Feature 3	600N/537E NE	Subsurface	Polar bear	cortical	-13,34	22,25	43,08	15,18	3,31	0,05	0,08	0,16	141
4886	17,4	QjJx-1	Feature 10	612N/518E SW	Subsurface	Bearded seal	cortical	-13,24	15,29	43,09	15,20	3,30	0,74	1,17	1,55	216
4891	15,9	QjJx-1	Feature 3	601N/537E NW	Subsurface	Polar bear	cortical	-13,08	20,99	42,78	15,18	3,29	0,13	0,19	0,20	124
4905	22,1	QjJx-1	Feature 3	380N/523E SW	Surface	Walrus	bit spongious but very clean	-14,56	11,15	42,42	14,96	3,31	0,57	0,83	1,09	116
4908	22,5	QjJx-1	Feature 74	378N/523E NE	Surface	Walrus	bit spongious but very clean	-14,61	11,24	42,02	14,80	3,31	0,79	1,33	1,92	88
4909	16,5	QjJx-1	Feature 74	378N/523E NE	Surface	Bearded seal	cortical	-13,29	14,58	44,89	15,40	3,40	0,85	1,45	1,70	119
4916	11,9	QjJx-1	Feature 74	376N/521E SE	Subsurface	Ringed seal	cortical	-14,47	18,09	42,13	14,56	3,37	0,46	0,76	0,89	131

Table B. δ^{66}Zn values of in house standard and reference material. Zn delta values are corrected for the standard JMC Lyon

	**Category**	**Material**	**n**	**δ^{66}Zn**	**SD**	**Expected value**	**Reference**
**AZE**	in house standard	bone	2	1.51	0.12	1.50 ‰ ± 0.04	17
						1.47 ‰ ± 0.11	18
**SRM 1486**	reference material	bone meal	4	1.22	0.06	1.17‰ ±0.07	18

Table C Results of the Kruskal-Wallis test ($\chi 2$ and p values) performed on the isotopic values (C, N and Zn) for the different species.

Kruskal-Walllis	χ^2	df	p-value
δ^{13}C	17.25	3	0.00063
δ^{15}N	36.00	3	7.37E-08
δ^{66}Zn	31.97	3	5.32E-07

Table D Matrix of the p-values resulting from the Nemenyi test comparing the isotope compositions of the different species.* p<0.05,** p<0.005, ***p<0.0005

$\delta^{13}C$			
	Polar Bear	Bearded Seal	Ringed Seal
Bearded Seal	0.22	-	-
Ringed Seal	0.69	0.84	-
Walrus	0.11	**0.00037*** **	**0.00566** **

$\delta^{15}N$			
	Polar Bear	Bearded Seal	Ringed Seal
Bearded Seal	**0.000029*** **	-	-
Ringed Seal	0.11	0.076	-
Walrus	**0.00000057*** **	0.33	**0.0019** **

$\delta^{66}Zn$			
	Polar Bear	Bearded Seal	Ringed Seal
Bearded Seal	**0.00000014*** **	-	-
Ringed Seal	**0.046***	**0.011***	-
Walrus	**0.017***	0.34	0.85

References

1. Dewing K, Turner EC. Structural setting of the Cornwallis lead-zinc district, Arctic Islands, Nunavut. Geol Surv Can Curr Res B-4. 2003; 1–9.

2. Wolff EW, Peel DA. Concentrations of cadmium, copper, lead and zinc in snow from near Dye 3 in south Greenland. Ann Glaciol. 1988;10: 193–197.

3. Boutron CF, Candelone J-P, Hong S. Greenland snow and ice cores: unique archives of large-scale pollution of the troposphere of the Northern Hemisphere by lead and other heavy metals. Sci Total Environ. 1995;160–161: 233–241. doi:10.1016/0048-9697(95)04359-9

4. Bruland KW. Oceanographic distributions of cadmium, zinc, nickel, and copper in the North Pacific. Earth Planet Sci Lett. 1980;47: 176–198.

5. Bruland KW, Orians KJ, Cowen JP. Reactive trace metals in the stratified central North Pacific. Geochim Cosmochim Acta. 1994;58: 3171–3182.

6. Bruland KW, Franks RP. Mn, Ni, Cu, Zn and Cd in the western North Atlantic. Trace metals in sea water. Springer; 1983. pp. 395–414. Available: http://link.springer.com/chapter/10.1007/978-1-4757-6864-0_23

7. Lohan MC, Statham PJ, Crawford DW. Total dissolved zinc in the upper water column of the subarctic North East Pacific. Deep Sea Res Part II Top Stud Oceanogr. 2002;49: 5793–5808.

8. Morel FMM, Price NM. The biogeochemical cycles of trace metals in the oceans. Science. 2003;300: 944–947.

9. Bermin J, Vance D, Archer C, Statham PJ. The determination of the isotopic composition of Cu and Zn in seawater. Chem Geol. 2006;226: 280–297.

10. Boyle EA, John S, Abouchami W, Adkins JF, Echegoyen-Sanz Y, Ellwood M, et al. GEOTRACES IC1 (BATS) contamination-prone trace element isotopes Cd, Fe, Pb, Zn, Cu, and Mo intercalibration. Limnol Oceanogr Methods. 2012;10: 653–665.

11. Zhao Y, Vance D, Abouchami W, De Baar HJW. Biogeochemical cycling of zinc and its isotopes in the Southern Ocean. Geochim Cosmochim Acta. 2014;125: 653–672.

12. Little SH, Vance D, Walker-Brown C, Landing WM. The oceanic mass balance of copper and zinc isotopes, investigated by analysis of their inputs, and outputs to ferromanganese oxide sediments. Geochim Cosmochim Acta. 2014;125: 673–693.

13. Darwent, C.M., 2001. High Arctic Paleoeskimo fauna: Temporal changes and regional differences. Ph.D. Dissertation, University of Missouri, Columbia, Missouri.

14. LeMoine G, Helmer J, Grønnow B. Late Dorset architecture on Little Cornwallis Island, Nunavut. Études/Inuit/Studies. 2003; 255–280.

15. Moore JJ, Hughen KA, Miller GH, Overpeck JT. Little Ice Age recorded in summer temperature reconstruction from vared sediments of Donard Lake, Baffin Island, Canada. J Paleolimnol. 2001;25: 503–517. doi:10.1023/A:1011181301514

16. Keen AJ, Udd JE. Mining in the Arctic. CRC Press; 1999.

17. Jaouen K. (2012) Les isotopes stables des métaux de transition (Cu, Fe, Zn) au service de l’anthropologie. Ecole Normale Supérieure de Lyon et Université Lyon 1.

18. Jaouen K., Beasley M., Schoeninger M. J., Hublin J.-J. and Richards M. Zinc isotope ratios of bones and teeth as new dietary indicators: results from a modern food web (Koobi Fora, Kenya). *submitted*.
