# Supplementary material for: Zinc Isotope Ratios as Indicators of Diet and Trophic Level in Arctic Marine Mammals
Source: PLoS One. 2016 Mar 24;11(3):e0152299. doi: 10.1371/journal.pone.0152299 (PMC4806842; doi:10.1371/journal.pone.0152299)
Supplement: S2 File — Table A. Standard reference materials used for calibration of δ13C relative to VPDB and δ15N relative to AIR. Table B. Standard reference materials used to monitor internal accuracy and precision. Table C. Accuracy and precision of calibration and check standards for each analytical. Table D. Accuracy and precision of calibration and check standards for all analytical sessions (cumulative). Table E. Duplicate sample carbon and nitrogen isotopic compositions and absolute difference between measurements. (PDF) [file pone.0152299.s002.pdf]

Supporting Information 2
Details on Calibration, Analytical Accuracy and Precision

Calibration

The following standard reference materials were used for calibration of δ^{13}C relative to VPDB and δ^{15}N relative to AIR (Table A).

Table A. Standard reference materials used for calibration of δ^{13}C relative to VPDB and δ^{15}N relative to AIR.

Standard	Material	Accepted δ^{13}C (‰, VPDB)	Accepted δ^{15}N (‰, AIR)
USGS40	Glutamic Acid	−26.389	−4.52
USGS41	Glutamic Acid	+37.626	+47.57

Accuracy and Precision

The following standards were used to monitor accuracy and precision (Table B). The isotopic compositions for these standards represent long-term averages based on the following number of analyses: n=195 (NIST 1577c), n=270 (SUBC−1), n=341 (MET).

Table B. Standard reference materials used for to monitor internal accuracy and precision.

Standard	Material	Accepted δ^{13}C (‰, VPDB)	Accepted δ^{15}N (‰, AIR)
NIST 1577c	Bovine liver	−17.51±0.10	+8.15±0.15
SUBC−1	Seal bone collagen	−13.67±0.11	+17.39±0.14
MET	Methionine	−28.60±0.08	−5.04±0.15

Table B summarizes the mean and standard deviation of carbon and nitrogen isotopic compositions for all check standards, as well as the standard deviation for all calibration standards for each analytical session associated with the data presented in this study – the mean of the calibration standard for an individual run is predetermined to calibrate the data.

Table C. Accuracy and precision of calibration and check standards for each analytical sessions.

Standard	Type	Run ID	*n*	δ^{13}C (‰, VPDB)	δ^{15}N (‰, AIR)
MET	Check	14-28	5	−28.60±0.03	−4.94±0.07
NIST 1577c	Check	14-28	2	−17.74±0.06	+8.07±0.02
SUBC−1	Check	14-28	6	−13.73±0.06	+17.30±0.06
USGS40	Calibration	14-28	8	±0.06	±0.17
USGS41	Calibration	14-28	9	±0.09	±0.20
MET	Check	14-40	4	−28.60±0.06	−4.92±0.12
NIST 1577c	Check	14-40	3	−17.54±0.10	+8.03±0.07
SUBC−1	Check	14-40	6	−13.64±0.11	+17.45±0.17
USGS40	Calibration	14-40	9	±0.06	±0.19
USGS41	Calibration	14-40	7	±0.22	±0.09
MET	Check	14-43	6	−28.58±0.10	−4.86±0.17
NIST 1577c	Check	14-43	4	−17.44±0.03	+8.31±0.16
SUBC−1	Check	14-43	6	−13.64±0.14	+17.51±0.05
USGS40	Calibration	14-43	7	±0.08	±0.06
USGS41	Calibration	14-43	6	±0.50	±0.18
MET	Check	14-46	7	−28.64±0.07	−4.95±0.25
NIST 1577c	Check	14-46	5	−17.61±0.10	+8.25±0.10
SUBC−1	Check	14-46	5	−13.74±0.02	+17.33±0.10
USGS40	Calibration	14-46	8	±0.03	±0.21
USGS41	Calibration	14-46	7	±0.18	±0.26
MET	Check	14-50	7	−28.61±0.06	−5.02±0.13
NIST 1577c	Check	14-50	5	−17.54±0.10	+8.14±0.17
SUBC−1	Check	14-50	6	−13.68±0.10	+17.44±0.17
USGS40	Calibration	14-50	7	±0.04	±0.15
USGS41	Calibration	14-50	8	±0.20	±0.15
MET	Check	14-32	7	−28.62±0.03	−5.00±0.18
NIST 1577c	Check	14-32	2	−17.77±0.01	+8.25±0.04
SUBC−1	Check	14-32	6	−13.71±0.04	+17.30±0.08
USGS40	Calibration	14-32	8	±0.02	±0.17
USGS41	Calibration	14-32	9	±0.14	±0.21

Standard	Type	Run ID	*n*	$\delta^{13}C$ (‰, VPDB)	$\delta^{15}N$ (‰, AIR)
MET	Check	15-02	5	−28.59±0.06	−5.06±0.10
NIST 1577c	Check	15-02	4	−17.48±0.08	+8.19±0.03
SUBC−1	Check	15-02	6	−13.75±0.11	+17.41±0.13
USGS40	Calibration	15-02	8	±0.05	±0.08
USGS41	Calibration	15-02	9	±0.13	±0.14
MET	Check	14-51	3	−28.68±0.01	−5.03±0.02
NIST 1577c	Check	14-51	4	−17.42±0.06	+8.19±0.07
SUBC−1	Check	14-51	6	−13.54±0.04	+17.52±0.10
USGS40	Calibration	14-51	8	±0.03	±0.25
USGS41	Calibration	14-51	5	±0.41	±0.31
MET	Check	15-07	6	−28.63±0.02	−5.05±0.11
NIST 1577c	Check	15-07	4	−17.50±0.09	+8.21±0.05
SUBC−1	Check	15-07	6	−13.67±0.05	+17.45±0.11
USGS40	Calibration	15-07	8	±0.03	±0.12
USGS41	Calibration	15-07	6	±0.27	±0.20
MET	Check	15-06	8	−28.58±0.06	−5.03±0.07
NIST 1577c	Check	15-06	6	−17.55±0.03	+8.21±0.15
SUBC−1	Check	15-06	6	−13.70±0.11	+17.37±0.04
USGS40	Calibration	15-06	8	±0.11	±0.07
USGS41	Calibration	15-06	8	±0.16	±0.26

Table C summarizes the mean and standard deviation of carbon and nitrogen isotopic compositions for all check standards, as well as the standard deviation for all calibration standards for all analytical sessions associated with the data presented in this study.

Table D. Accuracy and precision of calibration and check standards for all analytical sessions (cumulative).

Standard	Type	*n*	$\delta^{13}C$ (‰, VPDB)	$\delta^{15}N$ (‰, AIR)
MET	Check	58	−28.61±0.06	−4.99±0.15
NIST 1577c	Check	39	−17.54±0.11	+8.19±0.12
SUBC−1	Check	59	−13.68±0.10	+17.41±0.13
USGS40	Calibration	79	±0.05	±0.15
USGS41	Calibration	74	±0.24	±0.19

All samples were analyzed in duplicate. The mean difference between duplicate pairs was 0.06 for δ^{13}C and 0.11 for δ^{15}N. Values for sample pairs are listed in Table E as "A" and "B", respectively along with the absolute difference in the isotopic compositions between these measurements.

Table E. Duplicate sample carbon and nitrogen isotopic compositions and absolute difference between measurements.

SUBC	δ^{13}C A	δ^{13}C B	δ^{13}C Δ	δ^{15}N A	δ^{15}N B	δ^{15}N Δ
4695	-13.85	-13.90	0.06	19.32	19.16	0.16
4696	-13.66	-13.62	0.04	21.47	21.51	0.04
4702	-13.76	-13.83	0.06	21.14	21.21	0.07
4704	-13.64	-13.62	0.02	15.71	15.70	0.01
4711	-15.23	-15.20	0.04	10.92	10.94	0.02
4717	-13.78	-13.78	0.01	22.45	22.54	0.08
4719	-15.67	-15.78	0.11	11.38	11.20	0.17
4721	-13.59	-13.55	0.04	18.06	18.01	0.05
4722	-14.16	-14.15	0.00	24.41	24.57	0.16
4733	-12.05	-11.96	0.09	16.67	16.71	0.04
4735	-13.19	-13.17	0.02	14.04	14.05	0.00
4749	-14.94	-14.92	0.02	11.21	11.21	0.00
4753	-13.34	-13.34	0.00	15.08	15.23	0.15
4755	-13.51	-13.45	0.06	21.38	21.39	0.01
4757	-13.05	-13.12	0.05	15.45	15.47	0.02
4766	-13.39	-13.31	0.09	20.32	20.04	0.28
4767	-13.48	-13.52	0.04	18.02	18.11	0.09
4768	-13.27	-13.28	0.01	17.19	17.29	0.10
4773	-12.73	-12.75	0.02	20.73	20.79	0.06
4774	-15.13	-15.08	0.05	12.76	12.68	0.09
4775	-13.29	-13.41	0.12	15.42	15.41	0.01
4776	-12.27	-12.36	0.09	15.29	15.35	0.07
4787	-12.94	-12.88	0.05	17.62	17.58	0.04
4801	-12.58	-12.61	0.03	17.53	17.55	0.03
4808	-15.50	-15.57	0.07	11.27	11.02	0.25
4810	-12.43	-12.24	0.18	14.99	15.19	0.20
4811	-13.08	-13.03	0.05	15.41	15.58	0.17
4817	-12.68	-12.68	0.00	16.59	16.02	0.57
4823	-12.96	-12.90	0.07	14.10	14.05	0.05
4828	-13.30	-13.28	0.02	14.95	15.10	0.15
4829	-13.17	-13.27	0.09	15.25	15.20	0.05

SUBC	$\delta^{13}C$ A	$\delta^{13}C$ B	$\delta^{13}C$ Δ	$\delta^{15}N$ A	$\delta^{15}N$ B	$\delta^{15}N$ Δ
4835	-12.19	-12.12	0.07	15.82	15.96	0.14
4846	-13.97	-13.96	0.01	19.27	18.88	0.40
4849	-15.20	-15.22	0.03	11.52	11.37	0.15
4861	-14.51	-14.46	0.05	11.51	11.54	0.04
4864	-14.63	-14.50	0.13	11.60	11.61	0.01
4871	-14.72	-14.83	0.11	11.98	11.90	0.07
4875	-14.04	-13.97	0.08	24.05	24.38	0.34
4884	-13.36	-13.32	0.04	22.17	22.34	0.17
4886	-13.24	-13.17	0.07	15.29	15.22	0.07
4891	-13.13	-13.04	0.09	20.91	21.06	0.16
4905	-14.51	-14.61	0.10	11.17	11.13	0.04
4908	-14.63	-14.58	0.04	11.30	11.17	0.12
4909	-13.29	-13.34	0.05	14.58	14.78	0.20
4915	-15.59	-15.50	0.10	20.91	20.94	0.03
4916	-14.47	-14.46	0.01	18.09	18.13	0.04
